# The association between fear of missing out and social media addiction among university students: a mediation model with path-specific moderation

**DOI:** 10.3389/fpsyg.2026.1948452

**Published:** 2026-10-12

**Authors:** Rujie Luo, Ying Zhang, Lijian Lin, Ludan Zhang

**Affiliations:** School of Education Science, Guangxi Minzu University, Nanning, China

**Keywords:** fear of missing out, self-concept clarity, social comparison orientation, social media addiction, university students

## Abstract

**Introduction:**

This study investigated the relationship between fear of missing out (FoMO) and social media addiction among college students and its underlying mechanisms.

**Methods:**

Using a convenience sampling method, we administered an online questionnaire via the Wenjuanxing platform to university students from a specific region in China and obtained a total of 218 valid responses. This study employed a cross-sectional survey design. Participants completed the Fear of Missing Out Scale, the Bergen Social Media Addiction Scale, the Self-Concept Clarity Scale, and the Social Comparison Orientation Scale.

**Results:**

FoMO was significantly positively correlated with social media addiction and significantly negatively correlated with self-concept clarity. Self-concept clarity partially mediated the relationship between FoMO and social media addiction, with the indirect effect accounting for 45.7% of the total effect. Social comparison orientation significantly moderated the direct pathway from FoMO to social media addiction (B = 0.130, *p* = 0.005). The interaction between self-concept clarity and social comparison orientation was marginal (B = 0.086, *p* = 0.055) However, the index of moderated mediation was not significant (index = −0.060, 95% CI [−0.152, 0.066]), indicating that the indirect effect did not significantly vary as a function of social comparison orientation. Therefore, the moderating effect was path-specific rather than evidence of moderated mediation.

**Discussion:**

Higher social comparison orientation strengthened the direct positive predictive effect of FoMO on social media addiction, but marginally weakened the negative predictive effect of self-concept clarity on social media addiction. These findings suggest that FoMO is positively associated with university students’ social media addiction both directly and indirectly through its negative association with self-concept clarity, with social comparison orientation serving as a moderator of the direct pathway, though not of the entire mediating mechanism. The results offer theoretical support and practical implications for the prevention of and intervention in social media addiction among university students.

## Introduction

### The prevalence and significance of media addiction

In recent years, with the rapid development of the Internet in China, the number of Internet users in the country had reached 1.125 billion by December, 2025, corresponding to an Internet penetration rate of 80.1% (China Internet Network Information Center, 2026). By 2022, the number of social media users in China had grown to 1.108 billion, representing 96.7% of the total Internet user population (The Editorial Group of 2025 China Social Media Platform Guide Report, 2025). With the proliferation of digital technologies, social media platforms have become integral to daily life, particularly among college students, who constitute a significant demographic within these user bases. Although social media platforms (e.g., WeChat, Xiaohongshu, Douyin) have brought considerable convenience to our lives, excessive reliance on and use of social media may pose serious risks to individuals’ physical and mental health. Social media addiction refers to a compulsive pattern of social media use in which individuals are unable to voluntarily control their usage behaviors, devoting excessive time and energy to social media platforms, resulting in impaired social functioning, psychological well-being, and even physical health. Its core features include impulsivity of use, withdrawal symptoms, and loss of behavioral control. Research has indicated that the overall prevalence of social media addiction among social media users is approximately 24% (Cheng et al., 2021), manifesting as excessive and over-engaged use that significantly interferes with daily life (Ling et al., 2025), and exerting a range of adverse effects on emotional regulation, attentional control, and executive functioning (Ma and Liu, 2019). Previous studies have identified individual factors such as sense of belonging, social pressure (Wen et al., 2025), loneliness (Ling et al., 2025; Chen Y. F., 2024), and low self-esteem (Chen C., 2024; Chen Y. J., 2024) as important contributors to social media addiction. Existing research on the mechanisms underlying social media addiction has largely focused on external behavioral perspectives, such as interpersonal interaction and self-presentation. However, accumulating evidence from recent studies suggests that fear of missing out (FoMO) is one of the key psychological factors driving excessive social media use and addiction (Zhang et al., 2019). Although FoMO is widely recognized as a prevalent negative cognitive trait among contemporary college students and is closely associated with social media use behaviors, its intrinsic mechanisms in the development of social media addiction remain insufficiently explored and inadequately elucidated. Therefore, the present study takes FoMO as an antecedent variable to examine its influence on social media addiction, as well as the mediating and moderating mechanisms underlying this relationship.

In the present study, “social media addiction” is operationally defined as a problematic pattern of social media use characterized by impulsivity, withdrawal symptoms, and loss of behavioral control, leading to impairment in social, psychological, or physical functioning (Andreassen et al., 2017). We emphasize that this term refers to a dimensional tendency measured by the Bergen Social Media Addiction Scale (BSMAS), rather than a clinically diagnosed disorder. This conceptualization distinguishes our construct from generalized internet addiction or smartphone addiction, which encompass broader and more heterogeneous behaviors (Andreassen et al., 2017; Servidio et al., 2021).

### Fear of missing out and social media addiction

Given the detrimental effects of social media addiction on individuals, it is of considerable significance to explore its formation mechanisms and influencing factors. Fear of missing out (FoMO), also referred to as fear of missing out anxiety, is a diffuse composite emotional experience characterized predominantly by anxiety, accompanied by negative feelings such as fear, loss, worry, and frustration, which arises from the concern that one might miss important information or novel events. Its typical manifestations include a strong desire to know about others’ experiences and a persistent monitoring of external updates (Zhang et al., 2021a), as well as an urge to maintain continuous connections with others (Przybylski et al., 2013). According to self-determination theory, when individuals’ basic psychological needs are thwarted, they tend to exhibit maladaptive behavioral states. Existing research has demonstrated that unmet relatedness needs generate a yearning for belongingness, prompting individuals to seek sustained contact with others and to keep track of their activities, thereby giving rise to FoMO (Ma and Liu, 2019), along with the negative emotions it entails. In line with the compensatory internet use theory, individuals experiencing negative emotions such as anxiety and stress induced by FoMO may turn to social networking sites as a means of regulating these aversive emotional states (Ryan et al., 2016). Consequently, when FoMO levels are high, individuals tend to use social media applications to alleviate negative emotions (Xiong et al., 2022). Recent empirical evidence has also revealed that individuals with higher levels of FoMO use social media sites more frequently, and such intensive use increases the risk of social media addiction (Zhang et al., 2021b). Studies conducted by Wang et al. (2021) and Zhang et al. (2021b) suggest that the reason individuals with elevated FoMO levels become addicted to social media ultimately lies in the fulfillment of their psychological need to stay informed about their social surroundings. This psychological gratification generates a sense of physiological arousal, which, in turn, leads to a loss of control over social media use and eventual addiction. Based on the above theoretical reasoning and empirical findings, the present study proposes Hypothesis 1(H1). Fear of missing out positively predicts social media addiction.

### The mediating role of self-concept clarity

Building upon the established relationship between fear of missing out (FoMO) and social media addiction, it is particularly important to examine the underlying mechanisms that explain both how and when FoMO influences social media addiction. However, current understanding of this issue remains incomplete. Drawing upon the extant literature, the present study proposes that self-concept clarity may play a significant role in the relationship between FoMO and social media addiction. Self-concept clarity (SCC) is a positive personality trait reflecting the extent to which individuals hold clearly and confidently defined, internally consistent, and temporally stable self-beliefs (Campbell, 1990), it is crucial for ameliorating maladaptive behaviors. According to the self-discrepancy theory—and specifically its cognitive model of self-evaluation and emotional associations—individuals possess three core “self-domains”: the actual self, the ideal self, and the ought self. Inconsistencies among these self-representations are likely to engender negative emotions such as depression and anxiety (Higgins, 1987). Self-discrepancy theory, in particular, provides a cogent explanation for the pathway from FoMO to diminished self-concept clarity: the persistent anxiety about missing out on others’ rewarding experiences likely sustains a chronic discrepancy between individuals’ “actual self” and their “ideal self,” thereby depleting cognitive resources and undermining a coherent and stable self-perception. In prior empirical research on self-concept clarity, scholarly attention has focused primarily directed toward variables including social comparison orientation (An and Zhang, 2025), mindfulness (Liu et al., 2022), social exclusion (Wang X. J. et al., 2022), meaning in life (Chen and Wang, 2022), and self-esteem (Liu et al., 2017). Notably, problematic internet use and social media addiction have also been identified as significant correlates of individual self-concept clarity (Servidio et al., 2021). As a reflection of the degree to which individuals possess a clear understanding of themselves, higher self-concept clarity may render individuals less susceptible to external influences, thereby mitigating the propensity for social media addiction. In other words, FoMO—as a diffuse anxiety arising from concerns about missing out on rewarding experiences of others—is essentially a negative emotional state that requires regulation. According to self-discrepancy theory, this state may amplify the perceived gap between the actual and ideal selves, which in turn blurs self-concept. A compromised self-concept clarity then reduces individuals’ internal buffer against external social pressure, making them more vulnerable to compulsive social media use as a maladaptive coping mechanism (Servidio et al., 2021; Wang Y. et al., 2022). Drawing on the above theoretical integration, we propose that self-concept clarity serves as a key cognitive mechanism linking FoMO to social media addiction. On the basis of the above theoretical frameworks and empirical evidence, the present study proposes Hypothesis 2 (H2). Self-concept clarity mediates the relationship between FoMO and social media addiction.

### The moderating role of social comparison orientation

It is noteworthy that, although FoMO constitutes a significant predictor of social media addiction, not all individuals with high FoMO necessarily develop addictive social media use behaviors. This observation underscores the critical importance of identifying individual difference factor that amplify or attenuate the FoMO-induced social media addiction. In the present study, we conceptualize social comparison orientation as a risk-enhancing factor rather than a protective one, based on the tenets of Social Comparison Theory. Social Comparison Theory suggests that certain individual traits may exacerbate the adverse effects of risk factors (Festinger, 1954). For college students—particularly those who frequently experience FoMO—social comparison orientation, as a stable personality trait, may amplify the negative impact of FoMO (Lin and Jian, 2022). Social comparison orientation refers to the tendency of individuals, during social media use, to engage in comparisons with others in terms of abilities, personal achievements, physical appearance, and popularity, by browsing other users’ online self-presentations (e.g., personal photos, life updates) and their online interactions (Chen C., 2024; Chen Y. J., 2024). According to Social Comparison Theory, individuals need to establish a stable and clear understanding of themselves, typically relying on objective standards for self-evaluation; however, in the absence of such objective criteria, they tend to use others in the social environment as reference points for comparison (Heine et al., 2002). Social cognitive theory posits that individuals’ emotions and behaviors are influenced not only by external events but also by their cognitive appraisals (An and Zhang, 2025). Social comparison represents a critical factor in the formation of self-concept, and college students are particularly inclined to use social networking sites to acquire information about others, compare themselves with others, and use such comparisons as a basis for self-evaluation (Zuo, 2014). Specifically, individuals with a higher social comparison orientation are more likely to use others’ “highlight reels” as reference points for self-appraisal, which not only strengthens the eliciting effect of FoMO but also depletes individuals’ self-regulatory resources (Piko et al., 2025). Thus, high social comparison orientation is hypothesized to exacerbate the direct positive association between FoMO and social media addiction by intensifying the perceived social threat inherent in FoMO (Lin and Jian, 2022). Synthesizing the existing literature, the present study proposes that social comparison orientation may influence the relationships among FoMO, social media addiction, and self-concept clarity among college students, serving as a moderator in both the direct path and the latter half of the mediation pathway. On the one hand, social comparison orientation may amplify the negative effects of FoMO, a negative emotional state. Although FoMO is widely recognized as a core risk factor for social media addiction, there are substantial individual differences in the extent to which FoMO translates into addictive behaviors. Social comparison orientation, as a stable tendency to compare oneself with others in social contexts, may represent a key moderating variable that accounts for this variability. Social Comparison Theory (Festinger, 1954) maintains that individuals evaluate their own opinions, abilities, and social standing through comparisons with others. When individuals possess a high social comparison orientation, their attentional resources are continually directed toward others’ behaviors and states. This sustained comparative process transforms FoMO from a diffuse social anxiety into a concrete and palpable threatening cognition—for example, “I may indeed be missing out on something.” According to self-regulation theories of addiction, the essence of addictive behaviors lies in individuals’ systematic failure to regulate internal psychological states (e.g., emotions, stress, self-perception) (Li et al., 2023). In this self-regulatory failure, individuals gradually consolidate the use of a specific behavior (e.g., excessive social media use) as a means of rapidly alleviating discomfort, which progressively undermines self-control and ultimately culminates in a vicious cycle. Prior research has demonstrated that social comparison orientation significantly and positively predicts FoMO, and that FoMO fully mediates the relationship between social comparison orientation and compulsive social media use, suggesting that social comparison orientation may interact with FoMO to jointly influence social media addiction (Lin and Jian, 2022). Furthermore, Reer et al. (2019) found that social comparison orientation is closely associated with social media engagement, and that this relationship is partially mediated by FoMO. On the other hand, social comparison orientation may also moderate the relationship between self-concept clarity and social media addiction. According to Group-Value Theory, when individuals perceive themselves as accepted and valued by their group, they develop a stronger identification with and commitment to that group, thereby attaining a stable sense of self-worth (Lind and Tyler, 1988). Extending this logic, we argue that high social comparison orientation may weaken the protective function of self-concept clarity against social media addiction. When self-concept clarity is low, individuals lack a stable internal frame of reference for self-worth and consequently become more reliant on external social comparison information. Servidio et al. (2021) confirmed that self-concept clarity influences problematic smartphone use through the sequential mediation of social comparison orientation and FoMO, indicating that individuals with lower self-concept clarity are more susceptible to social comparison effects. In previous research, for individuals with high social comparison orientation, self-concept ambiguity prompts a strong reliance on social comparisons to “locate” themselves; conversely, for those with low social comparison orientation, even when self-concept is unclear, they are less likely to engage in frequent social comparisons to alleviate comparison-induced uncertainty. Consequently, compared with individuals with low social comparison orientation, those with high social comparison orientation are more vulnerable to the catalyzing effect of social comparison orientation in the pathway through which self-concept clarity influences social media addiction (Servidio et al., 2021). Thus, high social comparison orientation is expected to attenuate the negative predictive effect of self-concept clarity on social media addiction, thereby reducing the protective benefit typically conferred by a clear self-concept(Vogel et al., 2015; Piko et al., 2025) Nevertheless, scant empirical attention has been devoted to the moderating role of social comparison orientation in the relationships among FoMO, self-concept clarity, and the risk of social media addiction among college students. On the basis of the above theoretical reasoning and empirical findings, the present study proposes Hypothesis 3(H3). Social comparison orientation moderates the direct path from FoMO to social media addiction. We also exploratorily examined whether it moderates the second-stage path, but we did not hypothesize a moderated mediation effect on the entire indirect pathway.

### The present study

In sum, by integrating self-determination theory, and social comparison theory as the primary frameworks-and drawing on compensatory internet use theory and self-discrepancy theory as supplementary explanations for specific pathways, the present study constructed a mediation model with a moderator (see Figure 1) to simultaneously examine the relationships among FoMO, self-concept clarity, social comparison orientation, and social media addiction in college students. Specifically, this study aimed to elucidate the cognitive mechanisms and individual differences underlying college students’ social media addiction by investigating the mediating role of self-concept clarity and the moderating role of social comparison orientation in the relationship between FoMO and social media addiction. Although previous research has separately examined the roles of self-concept clarity and social comparison orientation in relation to social media use, the integrated mediation model with a moderator testing both pathways simultaneously remains largely unexplored. The present study fills this gap by answering both “how” (through self-concept clarity) and “under what conditions” (depending on social comparison orientation) FoMO is associated with increased social media addiction. The findings are expected to provide theoretical foundations and empirical evidence for guiding college students in critically evaluating online information, establishing stable self-cognition, and reducing excessive social media use and addictive behaviors.

**Figure 1:**
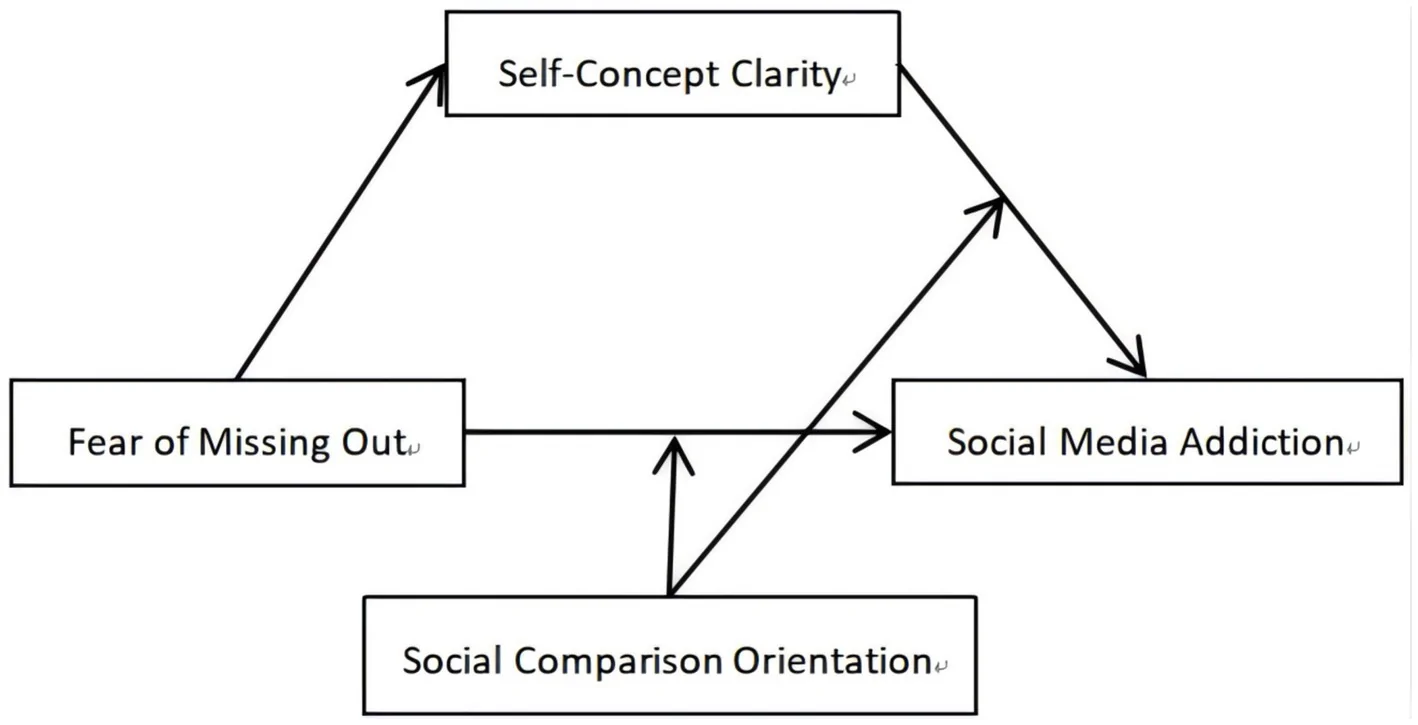
The conceptual model of the relationships among fear of missing out, self-concept clarity, social media addiction, and social comparison orientation.

## Materials and methods

### Participants

This study used a convenience sampling method to administer an online questionnaire via the Wenjuanxing platform to college students. Data were collected in February 2026. The survey link was distributed online through university student WeChat groups, QQ groups, and campus social media platforms, reaching college students from multiple universities across China. No monetary incentives were provided, but participants were informed that the results would contribute to academic research on college students’ social media use. To prevent duplicate responses, the Wenjuanxing platform’s IP address and device restriction functions were activated, and each participant was allowed to submit the questionnaire only once. A total of 300 questionnaires were distributed. Following rigorous screening criteria for psychological research, invalid responses were excluded based on the following criteria: (a) incomplete responses with missing items (n = 23); (b) excessively short (< 180 s) or excessively long (> 30 min) completion times (n = 35), suggestive of careless responding; (the 180-s threshold was set following common practices in prior research; e.g., Servidio et al., 2021, while completion times exceeding 30 min were considered indicative of interruption or distraction); and (c) patterned responding (e.g., selecting the same option for all items, (n = 12)) or contradictory responses to reverse-scored items (*n* = 12; i.e., responding in the same direction to both positively and negatively worded items, suggesting failure to read item content carefully). All exclusion criteria were pre-specified before data analysis. After screening, 218 valid questionnaires were retained, yielding an effective response rate of 72.6%. The final sample comprised 218 participants, including 56 males (25.7%) and 162 females (74.3%). In terms of academic level, 21 participants (9.6%) were first-year undergraduates, 32 (14.7%) were second-year undergraduates, 38 (17.4%) were third-year undergraduates, 78 (35.8%) were fourth-year undergraduates, and 49 (22.5%) were master’s students (doctoral students were not included in this category). With respect to academic majors, 82 participants (37.6%) majored in humanities and social sciences, 89 (40.8%) in science and engineering, 25 (11.5%) in arts, and 22 (10.1%) in other disciplines.

### Variables and measures

#### Fear of missing out

Fear of missing out was measured using the Fear of Missing Out Scale (FOMOS) developed by Przybylski et al. (2013). The scale consists of 10 items assessing FoMO (e.g., “I fear others have more rewarding experiences than me”). All items were rated on a 5-point Likert scale ranging from 1 (strongly disagree) to 5 (strongly agree), with higher scores indicating greater FoMO tendencies. In the present study, the Cronbach’s α coefficient for this scale was 0.89, indicating good internal consistency.

#### Social media addiction

Social media addiction was assessed using the Bergen Social Media Addiction Scale (BSMAS) developed by Andreassen et al. (2017). The scale comprises 6 items regarding social media use (e.g., “I spend a lot of time thinking about social media or planning how to use it”). Items were rated on a 5-point Likert scale ranging from 1 (very rarely) to 5 (very often), with total scores ranging from 6 to 30; higher scores reflect higher levels of social media addiction. In the present study, the Cronbach’s α coefficient for this scale was 0.87, indicating good internal consistency.

#### Self-concept clarity

Self-concept clarity was measured using the Self-Concept Clarity Scale (SCCS), originally developed by Campbell et al. (1996) and subsequently adapted by Niu et al. (2016). The scale consists of 12 items assessing self-concept (e.g., “Some of my views about myself often contradict each other”). Items 6 and 11 are reverse-scored. After recoding these items and computing total scores, higher total scores indicate greater self-concept clarity. Items were rated on a 5-point Likert scale ranging from 1 (strongly disagree) to 5 (strongly agree), with higher scores indicating greater self-concept clarity. In the present study, the Cronbach’s α coefficient for this scale was 0.88, indicating good internal consistency.

#### Social comparison orientation

Social comparison orientation was assessed using the social-media-specific version of the Iowa–Netherlands Comparison Orientation Measure (INCOM), originally developed by Gibbons and Buunk (1999) and subsequently adapted by Bai et al. (2013) for Chinese populations. This adapted version contains 12 items, with the wording contextualized to social media use (e.g., “In online social media, I often like to compare myself with those who perform better than me”). Items were rated on a 5-point Likert scale ranging from 1 (strongly disagree) to 5 (strongly agree), with higher scores indicating greater social comparison orientation. In the present study, the Cronbach’s α coefficient for this scale was 0.75, indicating acceptable internal consistency.

#### Data analysis

To examine the discriminant validity of the three core constructs (FoMO, SCC, SMA), we conducted confirmatory factor analysis (CFA) using item parceling. All 12 original SCC items were retained and used in the parceling procedure. The parceled indicators were then used as observed variables in the CFA. The same full SCC scale was used in the main mediation and moderation analyses; no SCC items were removed.

Data management, descriptive statistics, and correlation analyses were performed using SPSS 24.0. The PROCESS macro for SPSS (version 4.0, Hayes, 2013) was used to test the proposed hypotheses. All bootstrap analyses were conducted with 5,000 resamples and bias-corrected 95% confidence intervals. Specifically, we first examined the mediating role of self-concept clarity in the relationship between FoMO and social media addiction. Subsequently, we constructed interaction terms for FoMO × social comparison orientation and self-concept clarity × social comparison orientation to test the moderating effects of social comparison orientation on both the direct path and the mediation pathway. In subsequent analyses, gender, academic level, and academic major were included as control variables. Academic major was dummy-coded into three dummy variables (humanities and social sciences, science and engineering, and arts), with ‘other majors’ as the reference category. Gender was included as a covariate due to documented sex differences in social media use patterns and FoMO levels (Zhang et al., 2021a; Chen C., 2024; Chen Y. J., 2024). Academic level was included because developmental stage may influence self-concept clarity and social media dependence (Arnett, 2000; Servidio et al., 2021). Academic major was included because field of study may relate to differences in time spent on social media and peer influence that could confound the focal relationships.

## Results

### Common method bias test

Common method bias was assessed with Harman’s single-factor test. The results revealed seven factors with eigenvalues greater than 1, and the first unrotated factor accounted for 36.98% of the total variance, which is below the critical threshold of 40%. However, we acknowledge that Harman’s single-factor test is a relatively crude method for detecting common method bias and does not definitively rule it out (Podsakoff et al., 2003). To further address this concern, we implemented both procedural and statistical remedies. Procedurally, during the survey administration, we randomized the order of items across scales, assured participants of anonymity and confidentiality, and informed participants that there were no right or wrong answers to reduce social desirability bias. Statistically, in addition to the exploratory factor analysis, we conducted a confirmatory factor analysis (CFA) with a single-factor model, constraining all items to load onto one common factor. The single-factor CFA model showed poor fit: χ^2^/df = 3.074, CFI = 0.707, TLI = 0.690, RMSEA = 0.098, SRMR = 0.183, indicating that a single factor did not adequately account for the observed covariance among items. Taken together, these findings suggest that common method bias was not a serious concern in the present study.

### Descriptive statistics and correlation analysis

The descriptive statistics and intercorrelations among all variables are presented in Table 1. The mean BSMAS score in our sample (M = 18.18, SD = 5.60) was comparable to that reported in previous studies among Chinese college students (e.g., M = 17.85, SD = 5.42 in Zhang et al., 2021b), suggesting that the level of social media addiction in our sample was within the typical range for this population. FoMO was significantly and positively correlated with social media addiction and social comparison orientation. Social media addiction was significantly and positively correlated with social comparison orientation. Self-concept clarity was significantly and negatively correlated with both FoMO and social media addiction. Social comparison orientation was significantly and negatively correlated with self-concept clarity. In subsequent analyses, gender, academic level, and academic major were included as control variables.

**Table 1:** Means, standard deviations, and correlations among all variables (*N* = 218).

	*M*	*SD*	1	2	3	4	5	6	7
Gender	1.740	0.438	1						
Academic level	3.470	1.256	0.186^**^	1					
Academic major	1.940	0.946	0.007	−0.058	1				
FoMO	28.889	8.342	0.041	0.048	−0.027	1			
SMA	18.184	5.598	0.032	0.128	−0.128	0.717^***^	1		
SCC	35.151	9.354	−0.098	−0.089	0.099	−0.717^***^	−0.739^***^	1	
SCO	35.353	6.659	−0.106	−0.143*	−0.019	0.346^***^	0.298^***^	−0.264^***^	1

FoMO = fear of missing out; SMA = social media addiction; SCC = self-concept clarity; SCO = social comparison orientation. Gender was coded as 1 = male, 2 = female. Academic level was coded as 1 = first-year undergraduate, 2 = second-year undergraduate, 3 = third-year undergraduate, 4 = fourth-year undergraduate, 5 = master‘s student.

***p < 0.001; **p < 0.01; *p < 0.05.

### Discriminant validity and model comparison

To address the discriminant validity issue, we first examined the item-level CFA. The item-level model showed limited discriminant validity, in that the square roots of the AVE did not consistently exceed the inter-construct correlations. We therefore conducted CFA using item parceling based on all 12 original SCC items. The parceled three-factor model (FoMO, SCC, SMA) showed good fit: χ^2^(24) = 53.48, CFI = 0.982, TLI = 0.972, RMSEA = 0.075, SRMR = 0.030. All standardized loadings exceeded 0.80. The AVE values were 0.696 for FoMO, 0.801 for SCC, and 0.735 for SMA; the CR values were 0.872, 0.923, and 0.893, respectively. At the parceled level, the square roots of the AVE (FoMO = 0.834, SCC = 0.895, SMA = 0.857) were all greater than the corresponding latent inter-construct correlations (FoMO–SCC = −0.791, FoMO–SMA = 0.803, SCC–SMA = −0.827), satisfying the Fornell–Larcker criterion (Fornell and Larcker, 1981). The three-factor model also fit significantly better than a two-factor model (Δχ^2^ = 46.67, Δdf = 2, *p* < 0.001) and a one-factor model (Δχ^2^ = 88.63, Δdf = 3, *p* < 0.001). However, because the item-level CFA showed limited discriminant validity and the latent inter-construct correlations remained relatively high, the distinctiveness of the three constructs should be interpreted with caution. These results should therefore be regarded as providing acceptable, rather than strong, evidence for discriminant validity at the parceled level.

### Mediation analysis of self-concept clarity in the relationship between FoMO and social media addiction

To examine the mediating role of self-concept clarity in the relationship between FoMO and social media addiction, we employed Model 4 of the PROCESS macro for SPSS (Hayes, 2013), with gender, academic level, and academic major entered as control variables. The results (see Tables 2, 3) indicated that FoMO significantly and positively predicted social media addiction (B = 0.697, t = 14.481, *p* < 0.001). After the inclusion of the mediator variable, the direct predictive effect of FoMO on social media addiction remained significant (B = 0.378, t = 6.252, *p* < 0.001), and self-concept clarity significantly and negatively predicted social media addiction (B = −0.454, t = −7.467, *p* < 0.001). Furthermore, the bias-corrected bootstrap 95% confidence intervals for both the direct effect of FoMO on social media addiction and the indirect effect via self-concept clarity did not contain zero (see Table 3). These results demonstrate that FoMO not only directly and positively predicts social media addiction but also indirectly predicts social media addiction through the diminishment of self-concept clarity. The direct effect (0.378) and the indirect effect (0.319) accounted for 54.3 and 45.7% of the total effect (0.697), respectively. Given the cross-sectional nature of the data, this percentage should be interpreted with caution, as it may be unstable and should not be overinterpreted as evidence of causal mediation; the bootstrap confidence interval for the indirect effect provides a more reliable basis for inference (Hayes, 2013).

**Table 2:** Regression analysis for the mediation model (*N* = 218).

Outcome variable	Predictor	β	t	*p*
Social media addiction	Gender	−0.063	−0.557	0.578
	Academic level	0.071	1.852	0.065
	Major 1	0.238	1.431	0.154
	Major 2	0.049	0.284	0.776
	Major 3	−0.127	−0.618	0.537
	Fear of missing out	0.697	14.481	*p* < 0.001
*R*	0.733			
*R^2^*	0.534			
*F(df)*	*F* (5,212) = 60.999			
Self-concept clarity	Gender	−0.159	−1.390	0.166
	Academic level	−0.021	−0.538	0.591
	Major 1	−0.135	−0.806	0.421
	Major 2	−0.145	−0.837	0.403
	Major 3	0.253	1.221	0.224
	Fear of missing out	−0.703	−14.456	*p* < 0.001
*R*	0.721			
*R^2^*	0.529			
*F(df)*	*F* (5,212) = 57.437			
Social media addiction	Gender	−0.136	−1.334	0.184
	Academic level	0.062	1.801	0.073
	Major 1	0.177	1.190	0.235
	Major 2	−0.017	−0.112	0.911
	Major 3	−0.012	−0.066	0.947
	Self-concept clarity	−0.454	−7.467	*p* < 0.001
	Fear of missing out	0.378	6.252	*p* < 0.001
*R*	0.797			
*R^2^*	0.635			
*F(df)*	*F* (6,211) = 72.62			

FoMO = fear of missing out; SCC = self-concept clarity; SCO = social comparison orientation. Major 1 = humanities and social sciences; Major 2 = science and engineering; Major 3 = arts; all dummy variables were coded with “other majors” as the reference category. All continuous variables were standardized prior to analysis; thus, the coefficients reported are standardized coefficients (β).

**Table 3:** Direct, indirect, and total effects of FoMO on social media addiction via self-concept clarity.

Effect	Estimate	BootSE	BootCI lower	BootCI upper	Relative effect
Total effect	0.697	0.048	0.602	0.792	
Direct effect	0.378	0.061	0.259	0.498	54.3%
Indirect effect	0.319	0.070	0.200	0.469	45.7%

### Moderation analysis of social comparison orientation

Prior to interpreting the regression results, we examined the key assumptions of multiple regression. Multicollinearity was assessed using variance inflation factors (VIFs). As shown in the coefficients table, all VIFs were below 3.0 (range: 1.137–2.893), indicating that multicollinearity was not a serious concern (Field, 2013). Residual statistics revealed that the standardized residuals ranged from −2.507 to 3.678, with one case (case 36) showing a standardized residual of 3.678, slightly exceeding the conventional threshold of ±3.0. We examined this case and confirmed that it did not exert undue influence on the overall results, as all Cook’s distance values were below 1.0 (maximum Cook’s distance = 0.026). The Durbin-Watson statistic was 1.985, suggesting that residuals were independent. Residual plots (standardized residuals vs. standardized predicted values) suggested that the assumptions of linearity and homoscedasticity were reasonably satisfied. The normality of residuals was examined using a P–P plot, which indicated that the residuals were approximately normally distributed. These diagnostic checks confirmed that the regression assumptions were adequately met, supporting the validity of the reported findings.

To examine the moderating role of social comparison orientation, we employed Model 15 of the PROCESS macro for SPSS (Hayes, 2013), which corresponds to a mediation model with a moderator positing that both the direct path and the latter half of the mediation pathway are subject to moderation. This analytical framework was consistent with the theoretical model of the present study. Gender, academic level, and academic major were included as control variables. The results (see Table 4) indicated that the interaction between FoMO and SCO significantly predicted SMA (B = 0.130, SE = 0.046, *p* = 0.005, 95% CI [0.039, 0.220]). The interaction between SCC and SCO was marginal (B = 0.086, SE = 0.044, *p* = 0.055, 95% CI [−0.002, 0.173]). However, the index of moderated mediation was not significant (index = −0.060, SE = 0.051, 95% CI [−0.152, 0.066]), indicating that the indirect effect did not significantly vary as a function of SCO. Therefore, the findings do not provide evidence for moderated mediation. SCO significantly moderated the direct path, whereas the SCC × SCO interaction was only marginal and should be treated as exploratory. These path-specific moderating effects did not translate into a moderated mediation of the entire indirect effect. Further simple slope analyses were conducted (see Figures 2, 3). As illustrated in Figure 2, for participants with low social comparison orientation (M − 1SD), FoMO significantly and positively predicted social media addiction (simple slope = 0.23, t = 2.99, *p* < 0.05). For participants with high social comparison orientation (M + 1SD), FoMO also significantly and positively predicted social media addiction (simple slope = 0.49, t = 6.73, *p* < 0.001). These results indicate that the predictive effect of FoMO on social media addiction intensifies as individuals’ level of social comparison orientation increases.

**Table 4:** Moderated regression analysis for social media addiction.

Predictor	β	SE	t	*p*	95% CI
Gender	−0.085	0.100	−0.846	0.398	[−0.283, 0.113]
Academic level	0.067	0.034	1.971	0.050	[0.001, 0.133]
Major1	0.155	0.147	1.052	0.294	[−0.136, 0.446]
Major2	−0.013	0.152	−0.082	0.935	[−0.311, 0.287]
Major3	−0.030	0.183	−0.166	0.868	[−0.391, 0.330]
FoMO	0.355^***^	0.062	5.773	<0.001	[0.234, 0.477]
SCC	−0.470^***^	0.061	−7.742	<0.001	[−0.589, −0.350]
SCO	0.054	0.045	1.186	0.237	[−0.036, 0.143]
FoMO × SCO	0.130	0.046	2.812	0.005	[0.039, 0.220]
SCC × SCO	0.086	0.044	1.933	0.055	[−0.002, 0.173]
*R^2^*	0.645				
ΔR^2^(FoMO × SCO)	0.014^**^				
ΔR^2^(SCC × SCO)	0.006				
*F*	41.915^*^				

FoMO = fear of missing out; SCC = self-concept clarity; SCO = social comparison orientation. major1 = humanities and social sciences; major2 = science and engineering; major3 = arts; all dummy variables were coded with ‘other majors’ as the reference category. CI = bias-corrected bootstrap confidence interval (5,000 resamples).

***p < 0.001; **p < 0.01; *p < 0.05.

**Figure 2:**
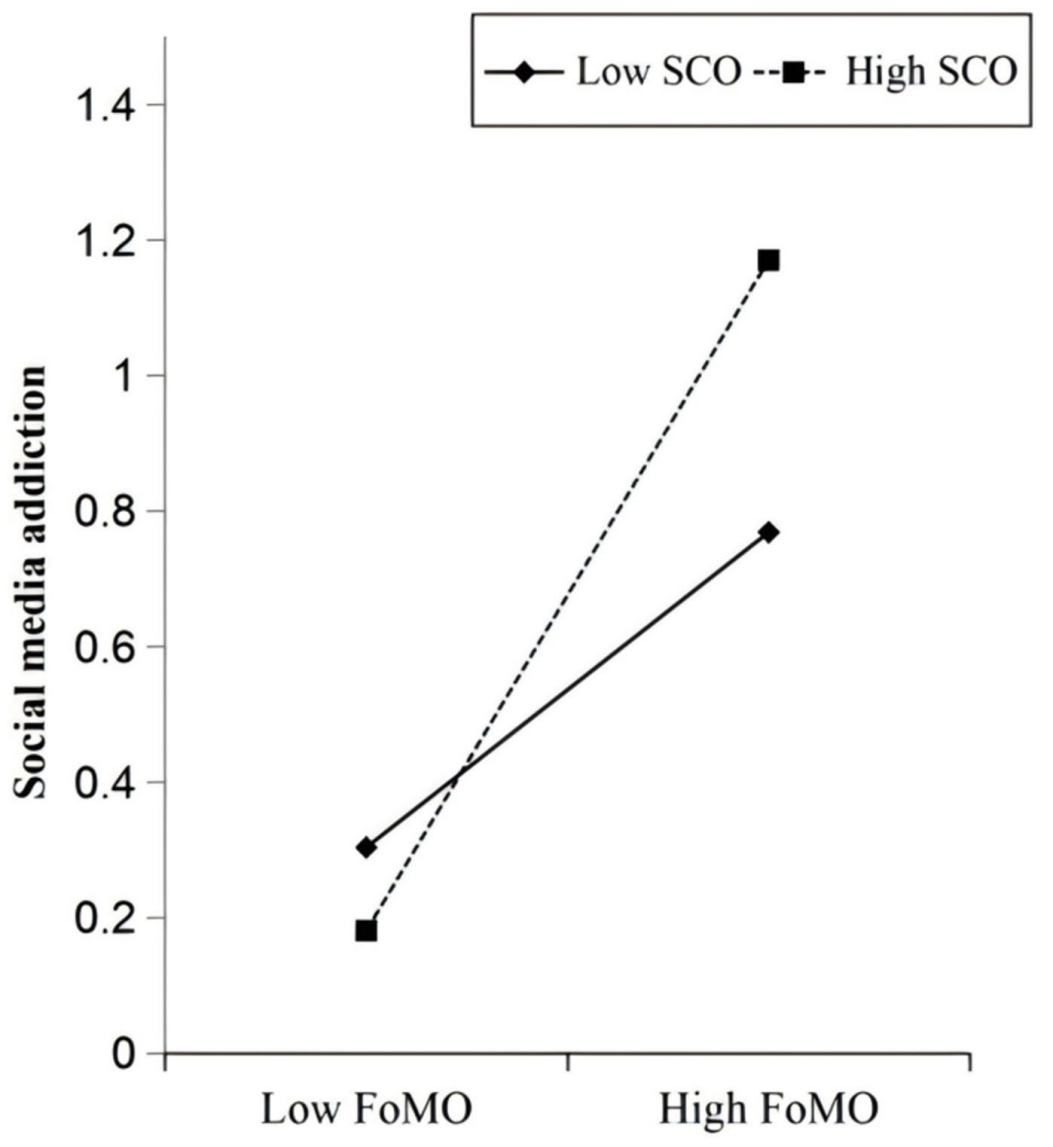
Interaction between fear of missing out and social comparison orientation in predicting social media addiction. The *x*-axis represents levels of fear of missing out (Low FoMO vs. High FoMO), and the two lines represent low and high social comparison orientation (SCO). The *y*-axis represents the predicted value of social media addiction.

**Figure 3:**
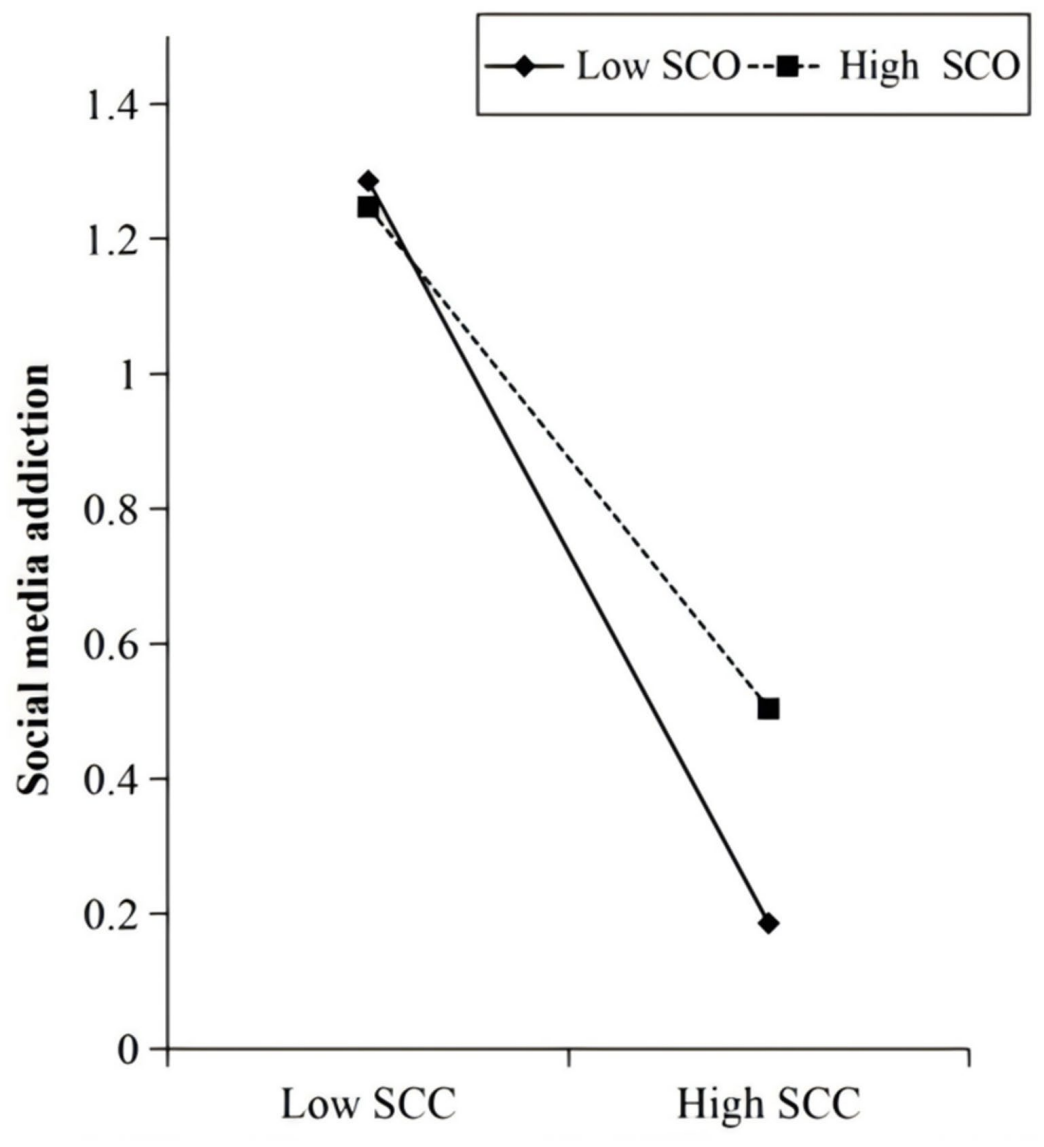
Interaction between self-concept clarity and social comparison orientation in predicting social media addiction. The *x*-axis represents levels of self-concept clarity (Low SCC vs. High SCC), and the two lines represent low and high social comparison orientation (SCO). The *y*-axis represents the predicted value of social media addiction.

As shown in Figure 3, for participants with low social comparison orientation (M − 1SD), self-concept clarity significantly and negatively predicted social media addiction (simple slope = −0.55, t = −7.01, *p* < 0.001). For participants with high social comparison orientation, self-concept clarity also significantly and negatively predicted social media addiction (simple slope = −0.37, t = −5.36, *p* < 0.001); however, this predictive effect was attenuated. These results suggest that as individuals’ level of social comparison orientation increases, the negative predictive effect of self-concept clarity on social media addiction tends to decrease. However, given that the index of moderated mediation was not significant (index = −0.060, 95% CI [−0.152, 0.066]), these simple slope results should be interpreted as path-specific moderation rather than evidence of moderated mediation.

Furthermore, across the three levels of social comparison orientation (M − 1SD, M, M + 1SD), the conditional indirect effects are presented in Table 5 for descriptive purposes only (see Table 5 note).

**Table 5:** Conditional indirect effects of FoMO on social media addiction via self-concept clarity at different levels of social comparison orientation.

Social comparison orientation	Effect	BootSE	BootCI lower	BootCI upper
Low *(M − 1SD)*	0.391	0.072	0.261	0.542
Mean	0.331	0.064	0.224	0.472
High *(M + 1SD)*	0.270	0.090	0.134	0.506
Index of moderated mediation	−0.060	0.051	−0.152	0.066

The index of moderated mediation was not significant (index = −0.060, 95% CI [−0.152, 0.066]). Therefore, the conditional indirect effects at different levels of SCO should not be interpreted as evidence of moderated mediation.

## Discussion

University students are a population at high risk for social media addiction. In this study, we selected university students as research participants to examine the association between FoMO and social media addiction and its underlying mechanisms. The results showed that FoMO was significantly and positively associated with social media addiction among college students. Self-concept clarity significantly mediated the relationship between FoMO and social media addiction. The present study found that SCO significantly moderated the direct path from FoMO to SMA (B = 0.130, *p* = 0.005), such that the positive association was stronger among individuals with higher SCO. The SCC × SCO interaction was marginal (B = 0.086, *p* = 0.055). However, the index of moderated mediation was not significant (index = −0.060, 95% CI [−0.152, 0.066]). These findings suggest that SCO moderates specific paths in the model—particularly the direct risk pathway—but does not significantly moderate the entire mediating mechanism. These findings provide empirical evidence for understanding the correlates and potential mechanisms underlying social media addiction among college students, and offer practical implications for preventing and intervening in addictive social media use in this population. However, given the cross-sectional design of this study, all findings should be interpreted as associations rather than causal relationships.

### The direct effect of FoMO on social media addiction

The study found that FoMO was positively associated with social media addiction among college students, which is consistent with previous research findings (Zhang et al., 2021a). Employing a cross-sectional design, this study identified FoMO as a significant risk factor for social media addiction. Self-determination theory emphasizes that when individuals’ basic psychological needs are thwarted, they tend to exhibit maladaptive behavioral states (Vansteenkiste and Ryan, 2013). The present results are in line with those of Wang and Wang (2021) and Zhang et al. (2021a, 2021b), suggesting that social media addiction may serve as a maladaptive coping strategy to alleviate the negative emotions associated with FoMO—in other words, FoMO may be associated with social media addiction through this coping mechanism. As a relational anxiety primarily stemming from sensitivity to social exclusion and a yearning for belongingness (Ali Al-Abyadh, 2025), FoMO reflects, from the perspective of self-determination theory, a state of frustrated basic psychological needs (Wang et al., 2021; Yuan et al., 2022). When real-life social interactions fail to adequately satisfy belongingness needs, social media emerges as a seemingly convenient compensatory pathway, offering low-threshold, high-frequency social comparison opportunities that enable individuals to continuously monitor others’ activities, thereby alleviating the anxiety arising from need frustration. This anxious state may reinforce dependence on social media, potentially contributing to social media addiction. Recent cross-cultural evidence has also supported the robust association between FoMO and problematic social media use. For instance, Aslan and Koç (2026) found that FoMO was a significant predictor of problematic social media use among Turkish adults, and that this relationship was moderated by psychological distress. This cross-cultural consistency suggests that the FoMO–social media addiction link may transcend cultural boundaries, although the specific mechanisms may vary across contexts.

### The mediating role of self-concept clarity

Consistent with the aforementioned hypothesis, the study found that self-concept clarity mediated the relationship between FoMO and social media addiction among college students. This result further enriches and extends the self-discrepancy theory of addiction, showing that FoMO is not only directly associated with university students’ social media addiction but also indirectly related to addiction through its negative association with individuals’ self-concept clarity (Higgins, 1987). Higher levels of FoMO were associated with lower self-concept clarity among college students, which in turn was associated with increased susceptibility to social media addiction. According to self-discrepancy theory, individuals’ self-system comprises three core domains—the actual self, the ideal self, and the ought self—and discrepancies among these self-representations engender negative emotional experiences such as depression and anxiety. Such discrepancies deplete cognitive resources, making it difficult for individuals to maintain coherent and stable self-knowledge, thereby reducing self-concept clarity. In line with self-regulation theories of addiction, college students are in a critical period of self-concept development and exhibit heightened emotional reactivity (Arnett, 2000), although this developmental characterization may be most applicable to younger undergraduate students (typically aged 18–22) and may not fully extend to older master’s students in our sample. The prevalence and use of social media expose them to considerable social comparison pressure. Multiple previous studies focusing on college students have found that those with lower self-concept clarity are more likely to develop social media addiction (Servidio et al., 2021; Zhang et al., 2025). When college students have low self-concept clarity, they lack a stable internal evaluative standard and are more susceptible to interference from information about others during social media use, tending to obtain external feedback through continuous refreshing, browsing, and interacting to affirm their self-existence and worth (Xiong et al., 2026). FoMO, as a diffuse anxiety arising from concerns about missing out on others’ rewarding experiences, inherently constitutes a negative emotional state. Given that college students are at a critical juncture for self-concept development, the level of self-concept clarity directly determines whether individuals can effectively cope with this anxiety. On the one hand, according to self-discrepancy theory, when individuals persistently experience FoMO, their attentional resources are substantially consumed by external comparisons, leaving insufficient capacity for stable self-definition and integration, which leads to a vague and contradictory self-concept (Chang, 2023). When self-concept is unclear, individuals’ behavioral decisions are more easily driven by external stimuli (e.g., immediate feedback from social media), resulting in compulsive usage patterns and ultimately addictive behaviors. On the other hand, according to self-determination theory, FoMO acts as a negative factor; as individuals’ FoMO levels increase, negative affect may intensify, which in turn may be linked to self-concept confusion, often manifesting as heightened inferiority and introversion. Under these circumstances, individuals may tend to employ virtual interactions and social media use as means of “substitute satisfaction” or “self-suppressive escape” to temporarily relieve discomfort (Wang Y. et al., 2022). This reliance behavior might eventually contribute to compulsive usage patterns, potentially manifesting as social media addiction.

### The moderating role of social comparison orientation

The study found that social comparison orientation significantly moderated the direct path. The moderating effect on the second-stage path (SCC → SMA) was only marginal (*p* = 0.055) and should be interpreted as exploratory. Importantly, the index of moderated mediation was not significant; therefore, these findings do not provide evidence that the indirect effect varies as a function of SCO. For the direct path, the relationship between FoMO and social media addiction was stronger among college students with high social comparison orientation. For the mediation pathway, high social comparison orientation marginally weakened the negative predictive effect of self-concept clarity on social media addiction. These findings indicate that social comparison orientation moderates specific paths in the model, but the index of moderated mediation was not significant. Importantly, our findings reveal that social comparison orientation does not exert a uniform moderating effect on different paths of the mediation model: it amplifies the direct risk pathway from FoMO to social media addiction, while simultaneously attenuating the protective function of self-concept clarity in the indirect pathway. This distinction is critical for understanding the complex role of social comparison in the development of social media addiction, and for designing targeted interventions.

First, compared with individuals with low social comparison orientation, the positive predictive effect of FoMO on social media addiction was stronger among college students with high social comparison orientation. A plausible explanation for this result is that individuals with high social comparison orientation exhibit greater vigilance and sensitivity to others’ behaviors and experiences. When FoMO is activated, high social comparison orientation individuals not only experience the anxiety of “fearing to miss out” but also tend to interpret this anxiety as a signal that “they are at a disadvantage in social comparisons.” This cognitive reappraisal further amplifies the emotional valence of FoMO, more strongly driving compensatory social media addictive behaviors, thereby contributing to social media addiction (Lin and Jian, 2022). Moreover, individuals with high social comparison orientation frequently engage in upward social comparisons, which continuously deplete self-regulatory resources, making FoMO more likely to directly drive social media addiction (Lin and Jian, 2022). Therefore, high social comparison orientation amplifies the positive predictive effect of FoMO on social media addiction, whereas low social comparison orientation buffers this risk, making FoMO less likely to directly drive addictive behaviors.

Second, in the latter half of the mediation pathway, social comparison orientation marginally moderated the negative predictive effect of self-concept clarity on social media addiction. Specifically, this moderating effect manifested as follows: compared with individuals with high social comparison orientation, those with low social comparison orientation exhibited a stronger negative predictive effect of self-concept clarity on social media addiction—that is, self-concept clarity was more effective in inhibiting social media addiction among low social comparison orientation individuals. In contrast, high social comparison orientation tended to weaken this protective function of self-concept clarity. This pattern suggests that social comparison orientation does not uniformly strengthen the indirect pathway; rather, it exerts divergent influences: it exacerbates the direct risk pathway while simultaneously attenuating the protective function of self-concept clarity in the indirect pathway (Servidio et al., 2021; Vogel et al., 2015). A plausible explanation for this result is that individuals with low social comparison orientation are less sensitive to external social information (particularly others’ behaviors and evaluations) and are less susceptible to self-concept threats from upward comparisons. Consequently, their self-concept clarity can stably fulfill behavioral guidance and emotional regulation functions, effectively resisting social media addiction. Conversely, individuals with high social comparison orientation are highly sensitive to information about others in social contexts (Gardner et al., 2002) and frequently engage in upward social comparisons, which readily expose them to perceived discrepancies between themselves and others, thereby triggering low self-esteem, self-doubt, and negative affect (Vogel et al., 2015). In this process, even if individuals possess a certain degree of self-concept clarity, this clarity is eroded or overshadowed by sustained social comparison pressure, rendering it difficult to effectively translate into inhibitory effects on social media use. In other words, high social comparison orientation, by continuously activating cognitive and emotional responses to “self–other” comparisons, undermines the buffering capacity of self-concept clarity against addictive behaviors, thereby impeding its protective function. This nuanced pattern—whereby social comparison orientation strengthens the direct association but marginally weakens the second-stage path (SCC → SMA)—highlights the complexity of the underlying mechanisms and underscores the need for differentiated intervention strategies targeting different risk pathways (Piko et al., 2025; Aslan and Koç, 2026). These interpretations are limited to path-specific moderation, as the overall moderated mediation index was not significant.

Moreover, recent person-centred research has suggested that problematic social media users may form distinct psychological profiles that differ in levels of FoMO, distress, and self-esteem (Aslan and Koç, 2026). This raises the possibility that the mediation and path-specific moderation patterns observed in the present study may not apply uniformly across all individuals, but rather may be more pronounced in certain subgroups (e.g., those with high FoMO and high social comparison orientation). Future research could employ person-centred approaches, such as latent profile analysis, to examine whether the present mediation model with a moderator operates differently across distinct user profiles.

To further examine the nature of these interaction effects, we conducted Johnson-Neyman analyses. The results showed that the effect of FoMO on social media addiction remained significant across the entire range of social comparison orientation (all ps < 0.05), with the effect size increasing as SCO increased. Similarly, the effect of self-concept clarity on social media addiction also remained significant across the entire range of SCO (all ps < 0.05), with the effect size decreasing. The Johnson-Neyman plots are presented in Figures 4, 5.

**Figure 4:**
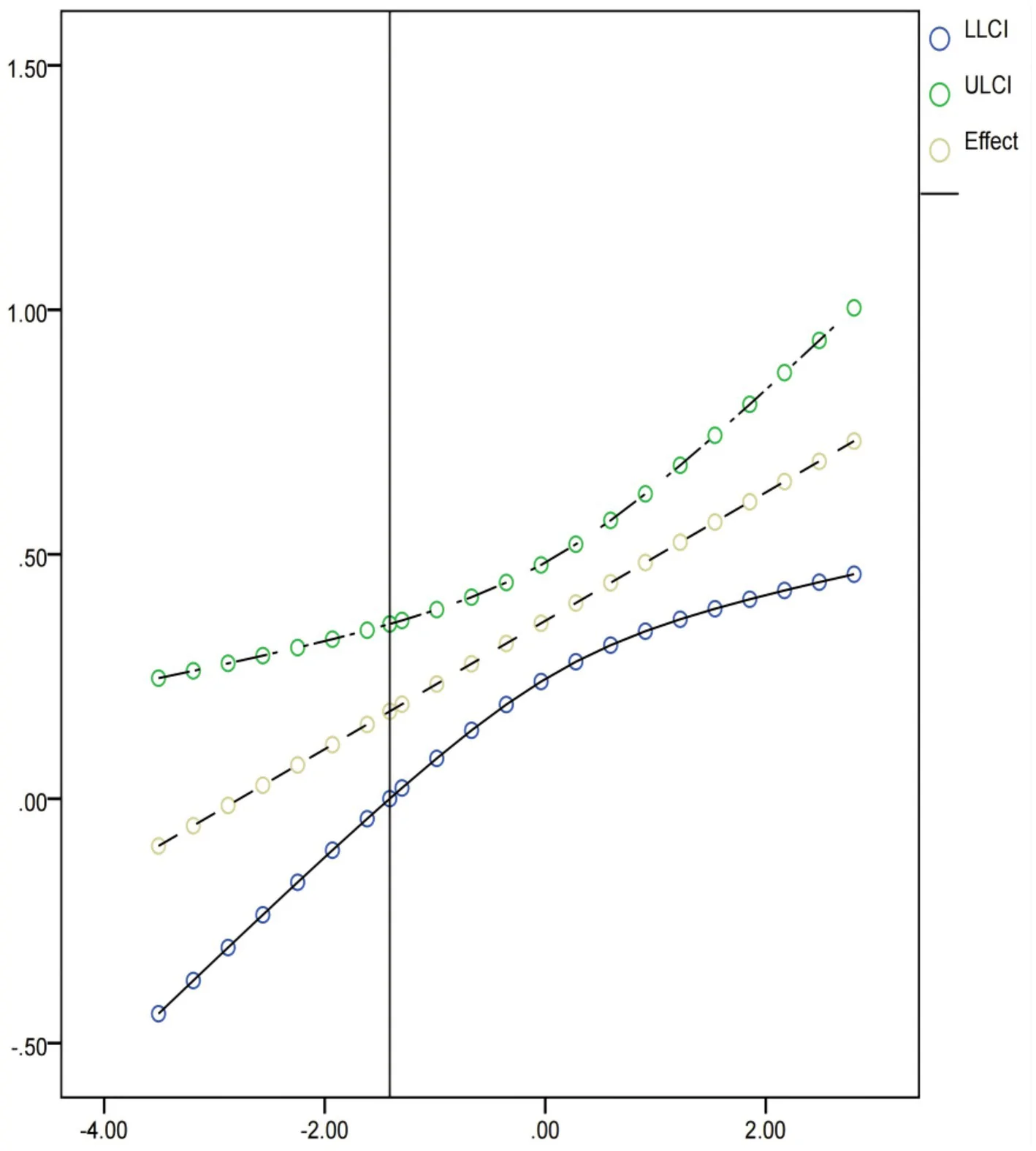
Johnson-Neyman plot for the interaction between fear of missing out and social comparison orientation in predicting social media addiction. The *x*-axis represents standardized values of social comparison orientation (SCO). The *y*-axis represents the conditional effect of FoMO on social media addiction. The solid line represents the conditional effect, and the dashed lines represent the 95% confidence intervals. The effect is significant when the confidence intervals do not cross zero.

**Figure 5:**
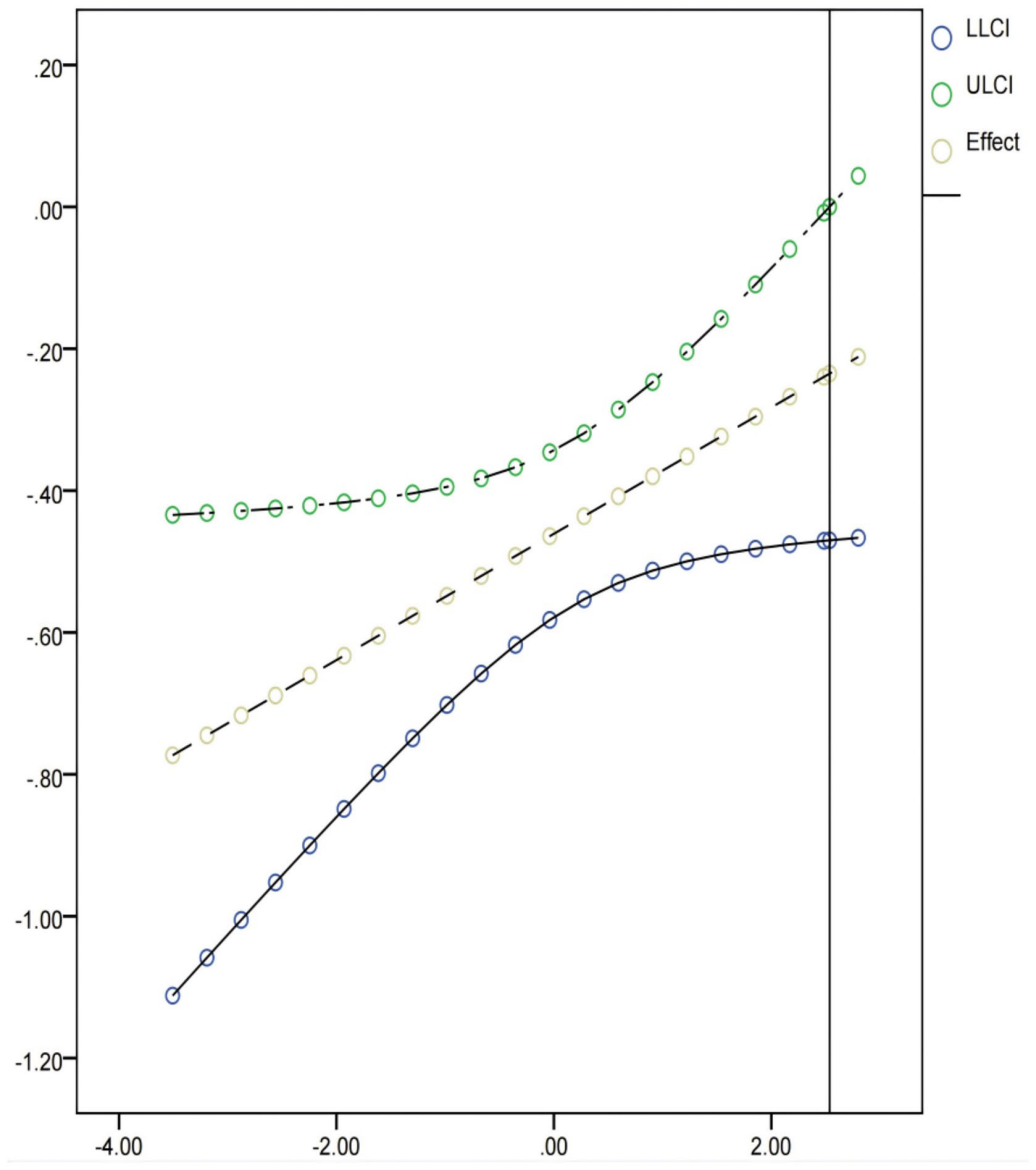
Johnson-Neyman plot for the interaction between self-concept clarity and social comparison orientation in predicting social media addiction. The *x*-axis represents standardized values of social comparison orientation (SCO). The *y*-axis represents the conditional effect of SCC on social media addiction. The solid line represents the conditional effect, and the dashed lines represent the 95% confidence intervals. The effect is significant when the confidence intervals do not cross zero.

### Summary of hypothesis testing

In summary, H1 was supported, as FoMO significantly and positively predicted social media addiction (B = 0.697, 95% CI [0.602, 0.792]). H2 was supported, as the indirect effect of FoMO on social media addiction via self-concept clarity was significant (indirect effect = 0.319, 95% CI [0.200, 0.469]). H3 was supported for the direct path: SCO significantly moderated the FoMO → SMA path (B = 0.130, *p* = 0.005). The SCC × SCO interaction was marginal (B = 0.086, *p* = 0.055) and exploratory. The index of moderated mediation was not significant (index = −0.060, 95% CI [−0.152, 0.066]), indicating that the indirect effect did not significantly vary across levels of SCO. Therefore, the moderating effects were path-specific rather than evidence of moderated mediation. However, given the marginal significance of the second-stage moderation and the non-significant index of moderated mediation, this support should be interpreted with caution.

## Implications and limitations

The study not only elucidates the cognitive mechanism (the mediating role of self-concept clarity) through which FoMO leads to social media addiction but also identifies individual differences in this cognitive mechanism (the moderating role of social comparison orientation). This mediation model with a moderator addresses both the question of how FoMO influences college students’ social media addiction and the question of under what conditions the direct predictive effect of FoMO on social media addiction and the mediating effect of self-concept clarity become more pronounced, thus contributing to a deeper and more nuanced understanding of the relationship between FoMO and individual psychological and behavioral adaptation.

The study also offers several practical implications for preventing FoMO-related social media addiction among college students. First, at the screening level, university counseling centers are encouraged to incorporate routine assessment of FoMO, social comparison orientation, and problematic social media use into their intake procedures, so as to identify at-risk students early and tailor subsequent interventions accordingly. Second, interventions should be differentiated based on individuals’ risk profiles. For students with high social comparison orientation, cognitive restructuring techniques can be employed to help them recognize and reappraise automatic comparison thoughts, particularly when triggered by peer content on social media. For students with elevated FoMO levels, group-based programs (e.g., emotion management and mindfulness-based stress reduction) may help them cultivate a stable sense of self-worth and reduce compulsive social media checking. More broadly, school-based psychoeducational initiatives can be delivered to modify general social comparison tendencies and thereby weaken the motivational link between FoMO and addictive use. Third, beyond clinical and counseling efforts, broader preventive strategies should be implemented. Social media literacy programs should incorporate content on selective self-presentation and the “highlight reel” nature of online content, helping students maintain a realistic perspective when viewing others’ posts. Students should also be taught behavioral self-regulation skills, including notification management, scheduled usage planning, and setting digital boundaries before bedtime. Additionally, career and personal development sessions can assist students in identifying their personal values, strengths, and offline sources of identity, thereby reducing their psychological reliance on social media validation. Fourth, it is important to note that these strategies are proposed as potential avenues rather than empirically validated interventions. Future research should test their efficacy through randomized controlled trial designs before they are implemented in real-world educational settings. Several limitations of the study should be acknowledged and addressed in future research. First, the sample size was relatively small; although it is comparable to those of previous studies examining similar mediation models with a moderator, we acknowledge that a formal power analysis was not conducted prior to data collection. Future studies should collect larger samples to enhance the robustness of the findings. Second, the study employed self-report measures for data collection, which may be subject to common method bias and social desirability effects, and the accuracy of participants’ self-perceptions may have influenced the results. Third, the cross-sectional design precludes causal inferences. Although our theoretical model, grounded in self-determination theory and social comparison theory, posits FoMO as a driver of social media addiction, we acknowledge that alternative directional models are equally plausible. For instance, excessive social media use may reciprocally exacerbate FoMO (Xiong et al., 2022), and low self-concept clarity may increase susceptibility to FoMO (Servidio et al., 2021). In addition, third-variable explanations cannot be ruled out; factors such as depression, anxiety, loneliness, self-esteem, and perceived social support may influence both FoMO and problematic social media use (Piko et al., 2025; Ling et al., 2025). However, the proposed directionality in our model is not arbitrary; it is supported by prior longitudinal studies (e.g., Zhang et al., 2021b) and theoretical frameworks demonstrating that FoMO precedes increased social media engagement (Przybylski et al., 2013). Future research should employ longitudinal designs or experimental paradigms to examine the causal relationships between FoMO and social media addiction, and to further explore the mechanisms through which self-concept clarity operates in the relationship between FoMO and social media addiction. In addition, several other limitations warrant mention. The sample was recruited through convenience sampling from multiple universities in a specific region of China, with a notable gender imbalance and without collecting participants’ age information or requiring active social media use as an inclusion criterion, which introduces self-selection bias and limits generalisability to broader college populations across regions or cultures. Moreover, the findings should be interpreted within the Chinese cultural context. As a collectivistic society that highly values social harmony and interpersonal relationships (Triandis, 1995), Chinese individuals may be particularly sensitive to social comparison information and peer opinions, which could amplify the effects of FoMO on social media use. In interdependent self-construals, self-concept is more closely tied to social relationships, potentially making self-concept clarity more vulnerable to disruption by social comparison processes (Campbell et al., 1996; Heine et al., 2002). However, because the present study did not directly measure cultural values or self-construals, future cross-cultural research is needed to examine whether the present findings generalize to other cultural contexts. Moreover, the single-source, single-time-point data collection and the use of adapted scales without validating their psychometric properties in the current sample raise additional concerns; we also relied on dimensional self-report scores rather than clinical diagnostic assessments. Furthermore, we did not control for important covariates, including social media usage time, platform types, and psychological factors such as depression, anxiety, loneliness, self-esteem, sleep, and social support, which may have led to overestimated associations. We also acknowledge that we did not test alternative directional models, and that our variable-centred approach assumes uniform applicability of the mediation model with a moderator, which may not hold across heterogeneous user profiles; recent person-centred research (e.g., Aslan and Koç, 2026) suggests that problematic social media users may form distinct psychological profiles, and future studies should adopt person-centred or mixture-modeling approaches to examine subgroup differences. Finally, the significant interaction effects we observed were relatively small and may be unstable, warranting cross-validation in independent samples.

The parceled CFA model showed acceptable fit and satisfied the Fornell-Larcker criterion. However, the item-level CFA showed limited discriminant validity, and the inter-construct correlations were relatively high. Therefore, the distinctiveness of the three constructs should be interpreted with caution. All 12 original SCC items were retained and used in both the parceling procedure and the main mediation and moderation analyses; no SCC items were removed. We did not report item-level AVE square roots and HTMT values in detail; future research should examine these indices more thoroughly. Future research should further examine the discriminant validity of these constructs using more refined measures and larger samples. Furthermore, the moderating effect of social comparison orientation on the second-stage path (SCC — SMA) was marginally significant (*p* = 0.055). This suggests that the buffering role of self-concept clarity against social media addiction under high social comparison orientation may be less robust than expected. The marginal significance may be partly attributable to the relatively modest sample size (*N* = 218), which may have limited statistical power to detect small interaction effects (Aiken and West, 1991). Given that interaction effects in psychological research often require larger samples to achieve adequate power, this finding should be interpreted as preliminary and exploratory. Importantly, because the overall index of moderated mediation was not significant, this marginal moderating effect does not constitute evidence of moderated mediation. Future studies with larger samples are needed to further clarify the nature and magnitude of this moderating effect.

Despite these limitations, our findings offer both theoretical and practical insights into preventing social media addiction among college students.

## Data Availability

The raw data supporting the conclusions of this article will be made available by the authors, without undue reservation.
